# Electric-field tunable Type-I to Type-II band alignment transition in MoSe_2_/WS_2_ heterobilayers

**DOI:** 10.1038/s41467-024-48321-1

**Published:** 2024-05-14

**Authors:** Jed Kistner-Morris, Ao Shi, Erfu Liu, Trevor Arp, Farima Farahmand, Takashi Taniguchi, Kenji Watanabe, Vivek Aji, Chun Hung Lui, Nathaniel Gabor

**Affiliations:** 1 Department of Physics and Astronomy, University of California, Riverside, CA 92521 USA; 2 National Laboratory of Solid State Microstructures, School of Physics, and Collaborative Innovation Center of Advanced Microstructures, Nanjing University, Nanjing, 210093 China; 3 Department of Physics, University of California, Santa Barbara, CA 93106 USA; 4 International Center for Materials Nanoarchitectonics, National Institute for Materials Science, 1-1 Namiki, Tsukuba, 305-0044 Japan; 5 Research Center for Functional Materials, National Institute for Materials Science, 1-1 Namiki, Tsukuba, 305-0044 Japan

**Keywords:** Electronic properties and materials, Optoelectronic devices and components

## Abstract

Semiconductor heterojunctions are ubiquitous components of modern electronics. Their properties depend crucially on the band alignment at the interface, which may exhibit straddling gap (type-I), staggered gap (type-II) or broken gap (type-III). The distinct characteristics and applications associated with each alignment make it highly desirable to switch between them within a single material. Here we demonstrate an electrically tunable transition between type-I and type-II band alignments in MoSe_2_/WS_2_ heterobilayers by investigating their luminescence and photocurrent characteristics. In their intrinsic state, these heterobilayers exhibit a type-I band alignment, resulting in the dominant intralayer exciton luminescence from MoSe_2_. However, the application of a strong interlayer electric field induces a transition to a type-II band alignment, leading to pronounced interlayer exciton luminescence. Furthermore, the formation of the interlayer exciton state traps free carriers at the interface, leading to the suppression of interlayer photocurrent and highly nonlinear photocurrent-voltage characteristics. This breakthrough in electrical band alignment control, interlayer exciton manipulation, and carrier trapping heralds a new era of versatile optical and (opto)electronic devices composed of van der Waals heterostructures.

## Abstract (web summary)

Photoluminescence and photocurrent measurements indicate that MoSe_2_/WS_2_ hetero-bilayers can be switched from type-I to type-II band alignment by applying a vertical electric field.

## Introduction

**Fig. 1:**
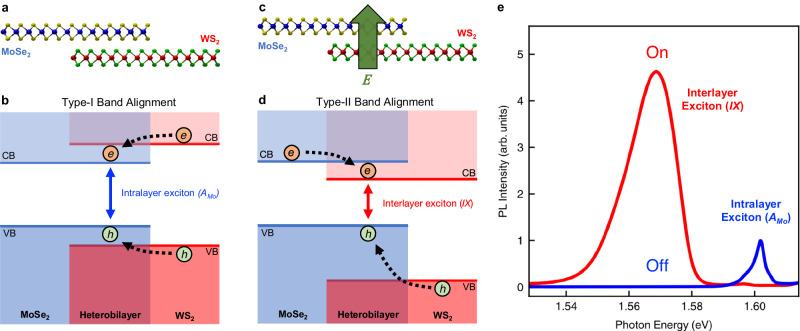
Illustration of electric-field-induced type-I to type-II band alignment transition in MoSe_2_/WS_2_ heterobilayer. **a**, **b** Atomic layers and energy band diagram of a MoSe_2_/WS_2_ heterobilayer with no electric field. The position-dependent conduction bands (CB) and valence bands (VB) of monolayer MoSe_2_ (blue) and WS_2_ (red) exhibit the type-I alignment. The photoexcited electrons and holes relax to the MoSe_2_ layer to form intralayer excitons (${A}_{{Mo}}$). **c**, **d** Atomic layers and energy band diagram under strong vertical electric field. The heterobilayer exhibits type-II band alignment. The photoexcited electrons (holes) relax to the WSe_2_ (MoSe_2_) layer to form interlayer excitons (${IX}$). **e** Measured photoluminescence (PL) spectra under zero (blue) and *E* = 0.25 V/nm (red) electric field. The interlayer excitons are turned on and off by the electric field.

Heterojunctions, where two different materials meet, form the fundamental building blocks for modern functional devices^1^, such as light emitting diodes^2^, photodetectors^3^ and field-effect transistors^4^. The critical determinant of heterojunction device characteristics lies in the alignment of conduction and valence bands between the two materials^5–7^. Type-I band alignment occurs when the band gap of one material is fully encompassed within the band gap of the other material, i.e., both the conduction band minimum (CBM) and the valence band maximum (VBM) of the heterostructure reside in the same material. This straddled band alignment causes photoexcited electrons and holes to relax into the same medium^8,9^ (Fig. 1a, b). As a result, the excitons have larger electron-hole wavefunction overlap, higher oscillator strength, and shorter radiation lifetime, which favor applications in light emitting devices^10,11^. In contrast, type-II band alignment situates the CBM of the heterostructure in one material and the VBM in the other (Fig. 1c, d). This staggered alignment causes the photoexcited electrons and holes to relax to different materials. This facilitates exciton dissociation and photocarrier extraction and hence favors applications in photodetection^12,13^ and photocatalysis^14^.

In the conventional scenario, the band energy offset between two materials is fixed; this imposes many constraints on device functionality. To usher in the next generation of devices, it is highly desirable to transition between different alignment types within a single heterostructure. The realization of tunable band alignment holds immense potential for unlocking unprecedented multifunctionality in device applications. However, achieving such demonstrations is challenging. While certain approaches involve material engineering through adjusting chemical compositions^15–18^ or layer thickness^19^, these methods necessitate multiple synthesis iterations, precise dopant control, or the use of different samples. A more efficient alternative envisions the capability to tune the band alignment types through straightforward electrical means in a single device, though, to the best of our knowledge, such a demonstration has not been realized.

A groundbreaking advancement in the fabrication of band-engineered heterostructures has emerged within the realm of two-dimensional (2D) van der Waals (vdW) materials^20–24^, notably including monolayer transition metal dichalcogenides (TMDs) like MoSe_2_ and WS_2_. These TMDs feature direct bandgaps, heavy carriers, robust excitons^25–32^, and innovative spin-valley-coupled physics^33–37^. In contrast to conventional grown heterostructures, 2D vdW heterostructures exhibit reduced dimensions, atomically sharp interfaces without dangling bonds, and high tolerance for lattice mismatch. These attributes greatly enhance their stability, tunability and overall functionality. Moreover, the continually expanding library of diverse 2D materials offers a rich palette of options for creating vdW heterostructures. Indeed, recent theoretical propositions have delved into the possibility of tuning band alignments in 2D vdW heterostructures through various means, including electric field^38,39^, strain^38^, interlayer spacing^38^, and twist angle^40^. However, experimental demonstration of such band alignment tuning is still lacking.

In this Article, we demonstrate an electric-field-induced transition between type-I and type-II band alignments in MoSe_2_/WS_2_ heterobilayers, as evidenced through photoluminescence (PL) and photocurrent (PC) spectroscopy. Figure 1 illustrates our key findings. Initially, in the absence of an external electric field, the heterojunction of monolayer MoSe_2_ and WS_2_ exhibits a type-I band alignment. In this state, the WS_2_ CBM is marginally higher than the MoSe_2_ CBM, while the WS_2_ VBM is considerably lower than the MoSe_2_ VBM (Fig. 1a, b). This configuration, with its straddled band gap, facilitates the relaxation of photo-excited electrons and holes to the same (MoSe_2_) layer, leading to a pronounced PL peak of intralayer excitons (${A}_{{Mo}}$) at 1.6 eV (blue line in Fig. 1e). However, the application of a strong vertical electric field, directed from the WS_2_ to MoSe_2_ layer, triggers a critical shift—the WS_2_ CBM moves below the MoSe_2_ CBM (Fig. 1c, d). This alteration leads to a type-II band alignment with staggered band gaps, where the photoexcited electrons and holes tend to relax to different layers—electrons to the WS_2_ layer and holes to the MoSe_2_ layer. This separation leads to the formation of interlayer excitons (${IX}$), which have a lower energy than their intralayer counterpart, resulting in a dominant PL peak at ~1.57 eV (Fig. 1e). Beyond the striking shift of the PL spectrum, the emergence of interlayer excitons also effectively traps electron-hole pairs at the heterojunction, thereby suppressing the interlayer photocurrent (*I*_pc_). This results in a highly nonlinear relationship between *I*_pc_ and the applied interlayer voltage. Overall, our findings highlight the profound impact of the type-I to type-II transition on the optical and optoelectronic properties of devices. The ability to electrically control this transition opens up exciting possibilities for designing innovative multifunctional devices using vdW heterostructures.

## Results and discussion

### Photoluminescence measurements

**Fig. 2:**
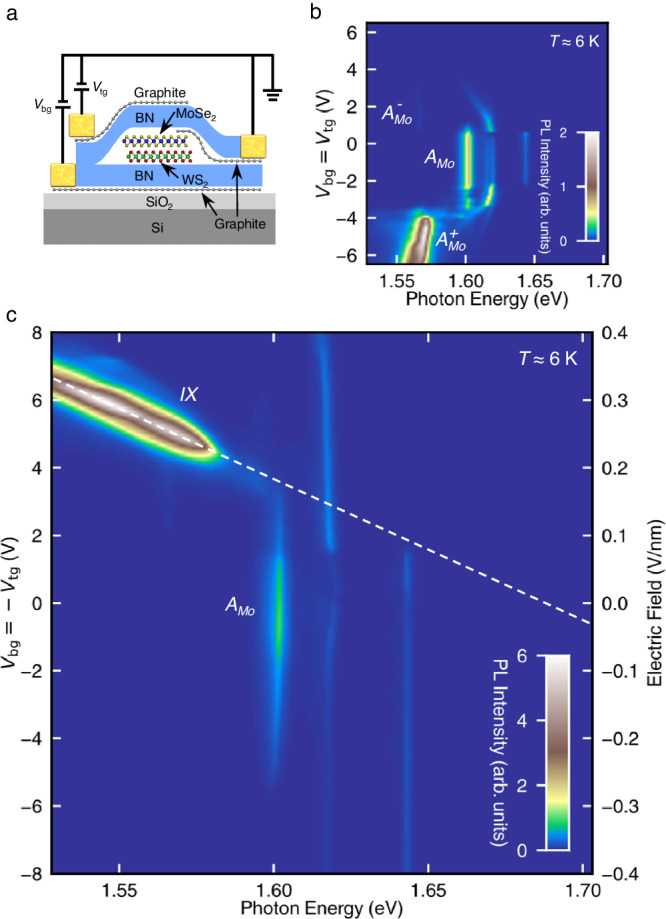
Photoluminescence signature of type-I to type-II band alignment transition in MoSe_2_/WS_2_ heterobilayers. **a** The schematic of a dual-gate MoSe_2_/WS_2_ heterobilayer device encapsulated by boron nitride. **b** The charge-density-dependent PL map of Device 1. Equal voltages *V*_bg_ = *V*_tg_ are applied to the bottom and top gates. The charge density is proportional to the gate voltages. The ${A}_{{Mo}}$, ${A}_{{Mo}}^{-}$, ${A}_{{Mo}}^{+}$ features arise from the intralayer excitons, electron-side and hole-side exciton polarons (or trions) in the MoSe_2_ layer, respectively. **c** The electric-field-dependent PL map of Device 1. Opposite voltages *V*_bg_ = −*V*_tg_ (left axis) are applied to the bottom and top gates to induce an interlayer electric field (right axis). An interlayer exciton (${IX}$) feature appears at high electric field. The dashed line is a linear extrapolation of its Stark shift to zero field. All measurements were performed with 532-nm laser excitation (incident power ≈ 3 μW) at sample temperature *T* ≈ 6 K.

Our experiment employs dual-gate MoSe_2_/WS_2_ heterobilayers encapsulated in hexagonal boron nitride (BN)^41^ (Supplementary Fig. S1, Fig. 2a). We use thin graphite flakes to contact the TMDs and electrodes to enhance device performance. The heterobilayers for PL measurements have twist angles of either ~$0^\circ$ or ~ $60^\circ$. Deviation from these angles will suppress the interlayer emission due to electron-hole momentum mismatch. Below we will present the PL results of Device 1 while the reflectance contrast results are presented in Section 4 of the Supplementary Information.

In Device 1, the top and bottom BN have similar thickness. By applying voltages with the same sign on the bottom gate (*V*_bg_) and top gate (*V*_tg_), we can inject carriers into the sample without inducing any vertical electric field. The charge-density-dependent PL map of Device 1 (Fig. 2b) exhibits a pronounced line (${A}_{{Mo}}$) at 1.60 eV at the charge neutrality region, which matches the reported A-exciton energy in BN-encapsulated MoSe_2_ monolayers^42–45^. Upon injecting electrons or holes into the heterobilayer, the ${A}_{{Mo}}$ peak subsides and two new PL peaks (${A}_{{Mo}}^{-}$, ${A}_{{Mo}}^{+}$) emerge at ~30 meV below the exciton on the electron and hole side, respectively. This energy separation is close to the known exciton-polaron (or trion) binding energies in monolayer MoSe_2_^42–45^. Therefore, we infer that ${A}_{{Mo}}$, ${A}_{{Mo}}^{-}$, ${A}_{{Mo}}^{+}$ originate from the intralayer exciton and exciton polarons in the MoSe_2_ monolayer. We note that the weak lines at higher energies than ${A}_{{Mo}}$ may arise from moiré effect or sample inhomogeneities, since they are not reproducible in other devices (Supplementary Figs. S8 and S9). Atomic reconstruction is unlikely to occur in this system due to the small moiré wavelength (~8 nm).

By applying voltages with opposite signs on the bottom and top gate (*V*_bg_ = −*V*_tg_), we can apply a vertical electric field between the MoSe_2_ and WS_2_ layers while maintaining the heterobilayer in the charge neutrality regime. Figure 2c displays the PL map of Device 1 at varying *V*_bg_ = –*V*_tg_ (left axis), from which we extract the out-of-plane electric field (right axis) with a BN dielectric constant of 3.4 (see the details in Supplementary Information, Section 2.1). At weak electric field (*E* < 0.16 V/nm pointing from WS_2_ to MoSe_2_), the ${A}_{{Mo}}$ PL line remains pronounced and exhibits no Stark shift; this observation confirms its intralayer nature and supports the type-I alignment of the heterobilayer (Fig. 1b). When the electric field exceeds 0.16 V/nm, the ${A}_{{Mo}}$ line subsides and below it emerges a new PL peak (${IX}$). The ${IX}$ peak redshifts linearly with a slope of 44 $\pm$ 11 meV per 0.1 V/nm of field; the $\pm$11 meV error is mainly due to the uncertain BN dielectric constant (2.6–4.2) with a minor $\pm$0.9 meV linear-fit uncertainty. At high field (*E* > 0.23 V/nm), ${IX}$ becomes bright and dominates the PL.

The Stark shift of ${IX}$ indicates that it has an out-of-plane dipole, a signature of interlayer excitons. By assuming that the electron and hole are localized in different layers, we deduce an electron-hole separation of 0.4 $\pm$ 0.1 nm based on the Stark shift. This separation is comparable to the interlayer spacing (~0.6 nm) of the heterobilayer^46^, providing evidence for the origin of interlayer excitons. When we extrapolate the Stark shift of ${IX}$ linearly to zero electric field, we arrive at an energy of ~1.69 eV, which is ~90 meV above the ${A}_{{Mo}}$ line at 1.60 eV. Considering the different exciton binding energies between ${A}_{{Mo}}$ and ${IX}$, we further estimate that the WS_2_ CBM resides ~40 meV above the MoSe_2_ CBM, comparable to a predicted 0.03-eV band offset in ref. ^47^ (see Supplementary Information, Section 3).

Our observation can be readily explained using the schematics in Fig. 1. At low field, the heterobilayer exhibits a type-I alignment with the WS_2_ CBM lying slightly above the MoSe_2_ CBM. Consequently, photocarriers relax to the MoSe_2_ layer to form intralayer excitons (Fig. 1a, b). As the electric field (directed from WS_2_ to MoSe_2_) increases, the WS_2_ CBM is lowered, leading to a transition to a type-II alignment. In the type-II configuration, photoexcited electrons and holes relax to different layers to form interlayer excitons (Fig. 1c, d). We note that an opposite electric field (from MoSe_2_ to WS_2_) elevates the WS_2_ CBM and does not induce the type-II alignment transition. This is consistent with our observation that no ${IX}$ peak appears at negative electric field (Fig. 2c).

### Photocurrent measurements

**Fig. 3:**
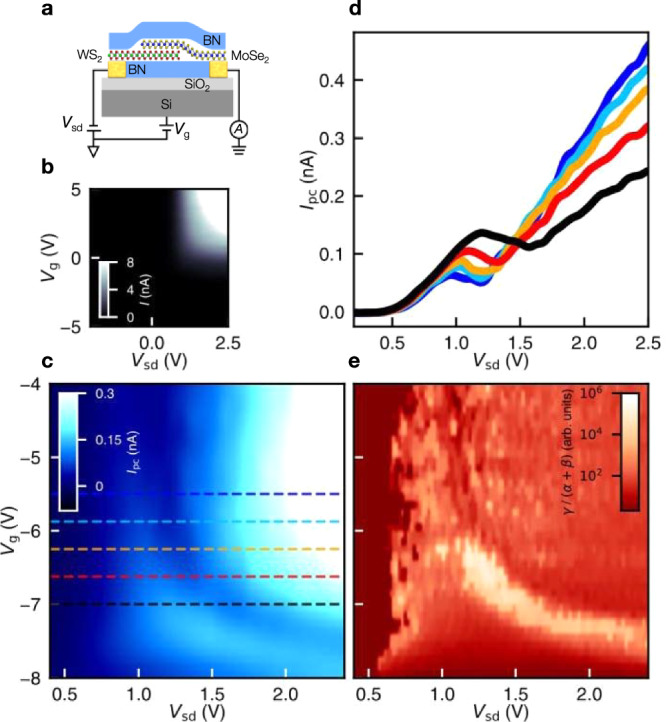
Photocurrent of MoSe_2_/WS_2_ heterobilayer. **a** The schematic of a MoSe_2_/WS_2_ heterobilayer device with silicon back gate (Device 2). **b** Grayscale map of interlayer current *I* as a function of the source-drain voltage (*V*_sd_) and gate voltage (*V*_g_) with no optical excitation. **c** Color map of interlayer photocurrent *I*_pc_ vs. *V*_sd_ and *V*_g_ and (**d**) Cross-cut *I*_pc_-*V*_sd_ profiles extracted from panel c at specific *V*_g_ values, denoted by dashed lines with corresponding colors. **e** Color map of the log of the relative rates of multi-particle decay (Auger processes) to single electron-hole pair decay $\gamma /(\alpha+\beta )$ as a function of *V*_sd_ and *V*_g_ over the same range as in (**c**).

In addition to the striking PL shift, the emergence of interlayer excitons can also drastically affect the optoelectronic charge transport through the MoSe_2_/WS_2_ interface. We have measured photocurrent in another BN-encapsulated MoSe_2_/WS_2_ heterobilayer (Device 2), which has source and drain contacts with a SiO_2_/Si back gate (Fig. 3a). We first characterize the device by measuring the interlayer current with no optical illumination as a function of source-drain voltage (*V*_sd_) and gate voltage (*V*_g_) at room temperature. *V*_sd_ is applied to the WS_2_ flake and current is measured from MoSe_2_ (Fig. 3a). The interlayer current is small at negative *V*_g_ and becomes increasingly large at positive *V*_g_ (Fig. 3b). This indicates *n*-type transport mediated by electrons in the conduction bands. At constant positive *V*_g_, the current is nearly zero at *V*_sd_ < 0, but increases dramatically with an exponential turn-on at *V*_sd_ > 0; such rectifying behavior is consistent with our band scheme in Fig. 1b, where the MoSe_2_ CBM is lower than the WS_2_ CBM. This further supports the intrinsic type-I alignment (see more discussions in Supplementary Information, Section 5.1).

Afterward we measure the interlayer photocurrent (*I*_pc_) under the excitation of an ultrafast laser. We tune the laser photon energy to be $\hslash \omega$ = 1.49 eV ($\lambda$ = 830 nm), which is close to the MoSe_2_ exciton resonant energy at room temperature (see Methods). Figure 3c displays a photocurrent color map at varying *V*_sd_ and *V*_g_. The photocurrent occurs predominantly in forward bias (*V*_sd_ > 0 V applied to WS_2_), consistent with the type I band alignment shown in Fig. 1b. Notably, the photocurrent map exhibits a narrow, curved region where *I*_pc_ drops with increasing *V*_sd_. Such an anomalous suppression of interlayer photocurrent can be seen clearly from the *I*_pc_-*V*_sd_ line traces at different *V*_g_ (Fig. 3d). As *V*_sd_ increases, the interlayer photocurrent first increases, then drops in the range of *V*_sd_ = 1–2 V, and afterward increases again. Similar non-monotonic *I*_pc_-*V*_sd_ characteristics are found for a wide range of *V*_g_ from −3.0 to −7.5 V, where the suppression region shifts from lower to higher *V*_sd_ values (Fig. 3c). This shift of suppression region with *V*_g_ is likely due to the change of contact resistance when the carrier density is modulated by the global silicon gate. A higher contact resistance means a higher *V*_sd_ is required to drive an equivalent interlayer voltage drop across the heterobilayer. By imaging the spatial photocurrent response, we confirm that this photocurrent suppression only occurs in the heterobilayer region (Supplementary Fig. S7).

We have extracted the interlayer electric field (*E*) from *V*_sd_ by modeling the device as a p-n junction and fitting its charge transport data (see Supplementary Information, Section 2.2). The anomalous suppression of photocurrent starts near *E* ~ 0.23 V/nm, comparable to the critical field (*E* ~ 0.16 V/nm) of interlayer exciton formation observed in the PL map (right axis in Fig. 2c). This suggests that the photocurrent suppression is induced by the interlayer exciton formation.

**Fig. 4:**
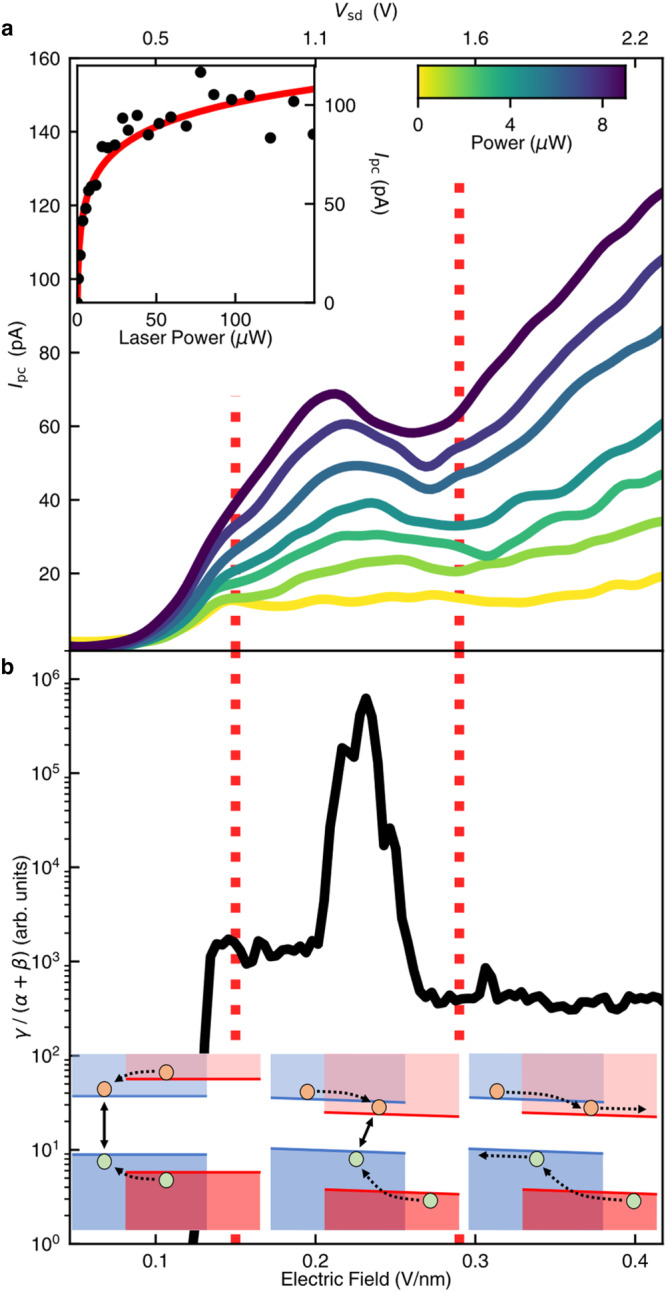
Photocurrent signature of type-I to type-II band alignment transition in MoSe_2_/WS_2_ heterobilayer. **a** Photocurrent (*I*_pc_) as a function of source-drain voltage (*V*_sd_, top axis) and interlayer electric field (bottom axis) under various excitation laser powers. The color of each curve represents the excitation power, as indicated by the color scale bar. The inset shows the photocurrent with increasing laser power at *V*_sd_ = 1.15 V (*E* = 0.24 V/nm). The red line is a fit based on the model described in the text. The gate voltage is *V*_g_ = −6.5 V in all measurements. **b** The best-fit $\gamma /(\alpha+\beta )$ value as a function of electric field. The vertical dashed lines approximately define three regions that correspond to the three scenarios depicted by the lower insets, which, from left to right, illustrate the absence, formation and dissociation of interlayer excitons at increasing field. The measurements were conducted at 830-nm excitation wavelength at room temperature.

To clarify the origin of the photocurrent suppression, we have investigated its dependence on the excitation laser power *P*. Figure 4a shows the photocurrent *I*_pc_ at varying *V*_sd_ (top axis) and electric field *E* (bottom axis) under increasing laser power at *V*_g_ = −6.5 V. At low laser power, *I*_pc_ increases monotonically with increasing *V*_sd_. At high laser power, however, *I*_pc_ drops in the range of *V*_sd_ = 1.0–1.5 V. As the laser power increases, the photocurrent suppression becomes more severe. By examining the power dependence of *I*_pc_ at varying *V*_sd_ and *V*_g_, we find that the photocurrent generally increases sublinearly with the laser power (e.g., see the inset of Fig. 4a) and, remarkably, the degree of sublinearity is closely related to the photocurrent suppression.

The photocurrent dynamics under pulsed excitation can be captured by using a simple model. Each laser pulse instantaneously generates ${N}_{0}$ electron-hole pairs, which drops to zero population before the next pulse arrives. The photocurrent is obtained as

1 $${I}_{{PC}}={ef}\alpha {\int}^{\infty}_{0} N\left(t\right){{{{{\rm{dt}}}}}}$$

Here $e$ is the elementary charge; $f$ is the pulse repetition rate; $\alpha$ is the carrier extraction rate; $N\left(t\right)$ is the time-dependent population of electron-hole pairs after the pulsed excitation. $N(t)$ decays according to the equation

2 $${dN}/{dt}=-(\alpha+\beta )N-\gamma {N}^{2}.$$

Here $\beta$ is the recombination rate of the electron-hole pairs. The $-(\alpha+\beta )N$ term describes the decrease of carrier population due to the extraction and first-order recombination of photocarriers. $\gamma$ is the decay rate due to exciton-exciton annihilation or Auger process. When exciton density *N* increases at increasing laser power, the nonlinear decay term ($-\gamma {N}^{2}$) becomes more important, leading to faster exciton decay and photocurrent suppression.

Solving Eq. (2) gives

3 $$N\left(t\right)=\frac{{{N}_{0}e}^{-\left(\alpha+\beta \right)t}}{1+\tfrac{\gamma {N}_{0}}{\alpha+\beta }\left(1-{e}^{-\left(\alpha+\beta \right)t}\right)}$$

which, when combined with Eq. (1), yields an analytical expression for the photocurrent under pulsed excitation:

4 $${I}_{{PC}}=\frac{{ef}\left(\alpha+\beta \right)}{\gamma }{{{{\mathrm{ln}}}}}\left(\frac{\gamma {N}_{0}}{\alpha+\beta }+1\right)$$

By assuming that ${N}_{0}$ scales linearly with the laser power (*P*), we can use Εq. (4) to fit the *I*_pc_—*P* data. The fitting is excellent for a broad range of *V*_sd_ and *V*_g_ values (e.g., see the line in the inset of Fig. 4a). From the fitting, we extract the relative rate $\gamma /\left(\alpha+\beta \right)$ (up to a proportionality constant) between multi-particle decay (Auger-like processes) and single electron-hole pair decay at different *V*_sd_ and *V*_g_ (Fig. 3e).

Figure 4b displays the best-fit $\gamma /\left(\alpha+\beta \right)$ value as a function of electric field at *V*_*g*_ = −6.5 V. We observe a striking peak of $\gamma /\left(\alpha+\beta \right)$ in the range *V*_sd_ = 1–1.5 V, which coincides with the *E*-field range where the photocurrent is suppressed in Fig. 4a. To consolidate this observation, we extract the $\gamma /\left(\alpha+\beta \right)$ value at varying *V*_sd_ and *V*_g_ (Fig. 3e) and compare it with the photocurrent map (Fig. 3c). The $\gamma /\left(\alpha+\beta \right)$ enhancement is found to coincide well with the photocurrent suppression. As the carrier extraction rate *α* and the Auger-like decay rate $\gamma$ typically evolve smoothly with electric field, the sharp peak of $\gamma /\left(\alpha+\beta \right)$ implies a sudden decrease of the electron-hole recombination rate $\beta$. This is consistent with the formation of interlayer excitons in a type-I to type-II transition because the interlayer excitons have much smaller recombination rate (longer lifetime) than the intralayer excitons due to the spatial separation of electrons and holes.

The insets of Fig. 4b illustrate how the interlayer exciton formation may account for the observed photocurrent behavior. At weak interlayer electric field (left inset), the heterobilayer has the type-I band alignment and hence exhibits rectifying behavior, in which the photocurrent increases monotonically with increasing interlayer field. When the electric field reaches a critical value *E*_c_ ~ 0.15 V/nm (comparable to *E*_c_ ~ 0.16 V/nm in the PL results in Fig. 2c), the heterobilayer transitions from type-I to type-II band alignment, enabling the formation of interlayer excitons (middle inset). The interlayer exciton formation traps the carriers at the interface, simultaneously suppressing the photocurrent and reducing the photocarrier decay rate $\beta$ (i.e. boosting $\gamma /\left(\alpha+\beta \right)$). When the increasing electric field becomes strong enough to dissociate the interlayer excitons (right inset), the exciton effect subsides and the photocurrent resumes its normal increasing trend with increasing *V*_sd_.

Besides Devices 1 and 2 presented above, we have also measured Devices 3 to reproduce the major PL results and Device 4 to reproduce both the major PL and photocurrent results (see Supplementary Information, Sections 1, 6 and 7).

In summary, we demonstrate controlled on/off switching of interlayer excitons in MoSe_2_/WS_2_ heterobilayers through a type-I to type-II transition, which substantially influences the optical properties and photocurrent behavior. This phenomenon stems from the closely aligned conduction band minima with field-tunable offset between monolayer MoSe_2_ and WS_2_, and it is not expected to occur in other TMD heterobilayers with large band offsets^47^. Our findings establish MoSe_2_/WS_2_ heterobilayers as a highly adaptable platform for excitonic research and applications. For instance, one may harness this effect for switching a hypothetical interlayer excitonic Bose-Einstein condensate, tuning exciton potential depth, and realizing depth-adjustable exciton traps within van der Waals heterostructure materials. The tuning mechanism complements other ‘live’ tunable parameters, such as strain, stress, and twist angles, and achieves ‘in-situ’ control of band-engineered exciton behaviors. This integration promises a new level of precision and adaptability in manipulating excitonic properties in these advanced materials.

## Methods

### Device fabrication

All MoSe_2_/WS_2_ heterobilayer devices are fabricated by applying a polycarbonate-based dry-transfer technique to stack different 2D crystals together. The substrates are silicon wafers with 300-nm-thick oxide layer. For the dual-gate Devices 1, 3, 4, we use a polycarbonate stamp to sequentially pick up a thin graphite flake (serving as the top-gate electrode), a thin BN flake (as the top-gate dielectric), monolayer MoSe_2_, monolayer WS_2_, a second thin graphite flake (as the contact electrode), another thin BN flake (as the bottom gate dielectric), and a third thin graphite flake (as the bottom-gate electrode). During the stacking process, we align the sharp edges of the MoSe_2_ and WS_2_ crystals so that the twist angles between them are expected to be close to 0° or 60°. Afterward, we deposit the stack of materials onto the Si/SiO_2_ substrate. Finally, we use the standard electron-beam lithography to deposit the gold contacts (70-nm thickness) onto the devices.

For single-gate Device 2 used in the photocurrent experiment, we first use electron-beam lithography to deposit the two gold contacts (as source and drain electrodes) on a Si/SiO_2_ substrate. Afterward, we use a polycarbonate stamp to transfer a BN flake to cover the area between the two electrodes. Upon this surface with pre-patterned electrodes, we transfer a MoSe_2_/WS_2_ heterobilayer stack by using a large thin BN flake to sequentially pick up monolayer MoSe_2_ and monolayer WS_2_. We align the sample position so that the WS_2_ layer contacts one electrode and the MoSe_2_ layer contacts the other electrode. This allows us to apply a bias voltage between the two layers.

### Photoluminescence experiments

The photoluminescence (PL) experiments are performed in a closed-cycle cryostat (Montana), where the sample temperature is estimated to be *T* ~ 6 K. The excitation light source is a 532-nm continuous-wave laser (Torus 532, Laser Quantum). The laser is focused onto the sample with a spot diameter of 1~2 μm by an objective lens (numerical aperture 0.6). The incident laser power is *P* ~ 3 μW. The PL is collected by the same objective and analyzed by a spectrometer (HRS-500-MS, Princeton Instruments) equipped with a charge-coupled-device (CCD) camera. Two Keithley K2400 source meters are used to independently control the top and bottom gate voltages.

### Photocurrent experiments

Photocurrent experiments are performed in vacuum in a customized Janis Research ST-3T-2 optical cryostat. Device 2 is measured at room temperature; Device 4 is measured at T = 50 K. The light source is an ultrafast Coherent Mira laser that generates pulses with 150-fs duration and 75-MHz repetition rate. The laser wavelength is tuned to either 790 nm or 830 nm, close to the optical band gap of monolayer MoSe_2_ as well as the energy of the interlayer exciton. The laser is focused onto the sample with a spot diameter of ~2 µm using a Thorlabs gradient-index (GRIN) lens. The laser position on the sample is controlled by a Thorlabs galvanometer. The galvo position, *V*_*sd*_, and *V*_g_ are controlled by two data acquisition cards (DAQs) from National Instruments. We measure the interlayer current with a pre-amplifier (DL Instruments 1211). The optically induced current is extracted from the total current by using a lock-in amplifier (Stanford Research) and optical chopper.

### Supplementary information

Supplementary Information

Peer Review File

## Data Availability

The data generated in this study have been deposited into https://github.com/qmolabucr/EField-Tunable-MoSe2WS2. This repository includes all the relevant data and the python scripts that are used to generate the figures such that the results can be fully replicated.
