# Supplementary material for: Electric-field tunable Type-I to Type-II band alignment transition in MoSe2/WS2 heterobilayers
Source: Nat Commun. 2024 May 14;15:4075. doi: 10.1038/s41467-024-48321-1 (PMC11093968; doi:10.1038/s41467-024-48321-1)
Supplement: Supplementary file 1 — Supplementary Information [file 41467_2024_48321_MOESM1_ESM.pdf]

Supplementary Information of
"Electric-field tunable Type-I to Type-II band alignment transition in $MoSe_2/WS_2$ heterobilayers"

Jed Kistner-Morris[1]*, Ao Shi[1]*, Erfu Liu[1,2], Trevor Arp[1,3], Farima Farahmand[1], Takashi Taniguchi[4], Kenji Watanabe[5], Vivek Aji[1], Chun Hung Lui[1†], Nathaniel Gabor[1†]

[1]Department of Physics and Astronomy, University of California, Riverside, CA 92521, USA
[2]National Laboratory of Solid State Microstructures, School of Physics, and Collaborative Innovation Center of Advanced Microstructures, Nanjing University, Nanjing, 210093, China
[3]Department of Physics, University of California, Santa Barbara, CA 93106, USA
[4]International Center for Materials Nanoarchitectonics, National Institute for Materials Science, 1-1 Namiki, Tsukuba 305-0044, Japan
[5]Research Center for Functional Materials, National Institute for Materials Science, 1-1 Namiki, Tsukuba 305-0044, Japan

1. Optical images of $MoSe_2/WS_2$ heterobilayer devices 2
2. Calculation of the interlayer electric field 2
2.1. Electric field in Devices 1, 3, 4 2
2.2. Electric field in Device 2 3
3. Estimation of the conduction band minimum offset 4
4. Additional results of Device 1: Reflectance contrast measurements 5
5. Additional results of Device 2 7
5.1. Dark current characteristics 7
5.2. Comparison of heterobilayer and monolayer photocurrent response 7
6. Results of Device 3: Reproducing interlayer exciton PL 8
7. Results of Device 4: Photoluminescence and photocurrent 9
7.1. PL measurements 9
7.2. Photocurrent measurements 10
Supplementary References 12

1. Optical images of $MoSe_2/WS_2$ heterobilayer devices

We have measured the photoluminescence (PL) in Devices 1, 3, 4, and the photocurrent in Devices 2, 4. Devices 1, 3, 4 have dual gates and Device 2 has a single gate. Figure S1 shows the optical images of Devices 1 – 4.

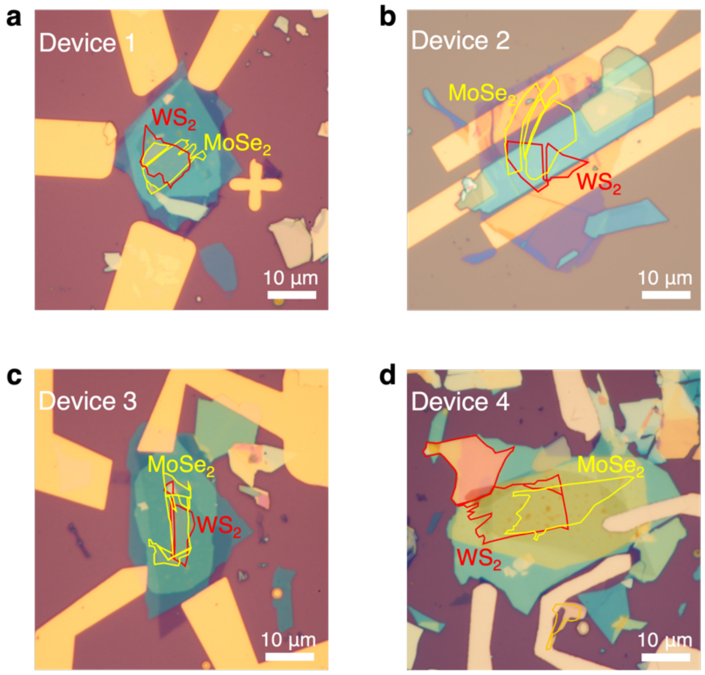

Figure S1 | a-d, Optical images of Devices 1 – 4.

2. Calculation of the interlayer electric field

2.1. Electric field in Devices 1, 3, 4

We determine the vertical electric field in the dual-gate $MoSe_2/WS_2$ heterobilayer devices by using a simple model of electrostatic capacitors. The bottom and top boron nitride (BN) flakes in our dual-gate devices have similar thickness. Therefore, by applying voltages of equal magnitude but opposite signs to the bottom and top gates ($V_{bg} = -V_{tg}$), we can induce an interlayer electric field while keeping the sample approximately charge neutral. The strength of the electric field can be calculated from the voltage difference $\Delta V = V_{bg} - V_{tg}$.

Figure S2 displays the electrostatic geometry of a $MoSe_2/WS_2$ heterobilayer with thickness d, which is sandwiched between the top and bottom BN with thickness d_1 and d_2, respectively. The static dielectric constants of BN and the heterobilayer along the vertical direction are denoted as $\varepsilon_{\mathrm{BN}}$ and $\varepsilon_{\mathrm{bi}}$, respectively. The electric fields across the top BN, the heterobilayer, and the bottom BN are denoted as E_1, E, and E_2, respectively. The voltage difference (ΔV) between the bottom and top gates is related to the electric fields as

$$\Delta V = d_1 E_1 + dE + d_2 E_2 \tag{1}$$

As the dual gates inject no net charge into the heterobilayer, we use simplified boundary conditions at the two interfaces:

$$\varepsilon_{\mathrm{BN}} E_1 = \varepsilon_{\mathrm{bi}} E = \varepsilon_{\mathrm{BN}} E_2 \tag{2}$$

Combining Eq. (1) and (2), we obtain

$$E = \frac{\Delta V}{\frac{\varepsilon_{\mathrm{bi}}}{\varepsilon_{\mathrm{BN}}}(d_1+d_2)+d} \tag{3}$$

In our calculation, we use d = 1.2 nm for the $MoSe_2/WS_2$ heterobilayer, $\varepsilon_{\mathrm{bi}} \approx \varepsilon_{\mathrm{MoSe_2}} = 4.4$ for the heterobilayer, and $\varepsilon_{\mathrm{BN}} = 3.4$ for BN, which are consistent with the literature[1–5]. For Device 1, we use $d_1 = d_2 = 15$ nm for the top and bottom BN. The calculated electric field for varying ΔV is shown in the right axis of Figure 2c of the main paper. For Device 3 presented in Figure S8, we use $d_1 = d_2 = 15$ nm for the top and bottom BN. The calculated electric field is shown in the right axis of Figure S8b.

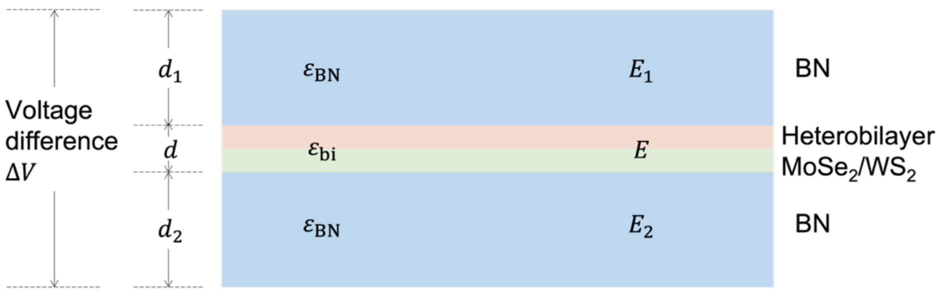

Figure S2 | The electrostatic geometry of the dual-gate heterobilayer devices.

2.2. Electric field in Device 2

For Device 2 used in the photocurrent experiment, the application of a source-drain voltage V_{sd} can induce an electric field (*E*) between the two layers. We can extract the interlayer electric field by using a simple model of p-n junction, whose current is expressed by the following equation for ideal diode:

$$I = I_0\left(e^{\frac{eV_D}{k_B T}} - 1\right) \tag{4}$$

Here V_D is the voltage drop between the two layers; I_0 is the saturation current at large negative V_D. Generally, V_D differs from the source-drain voltage V_{sd} due to the existence of contact resistance and in-plane sample resistance. Their ratio $n = V_D/V_{sd}$ is called the ideality[6]. When $eV_D \gg k_B T$, we can neglect the -1 term in Eq. (4) and express the current as:

$$I = I_0\, e^{\frac{enV_{sd}}{k_B T}} \tag{5}$$

We can use Eq. (5) to fit the $I - V_{sd}$ data in our experiment at low V_{sd} and obtain the best-fit ideality value n at a certain gate voltage (see the illustration in Figure S3). We assume that n remains constant from low to high V_{sd}, and calculate the interlayer electric field as:

$$E = \frac{V_D}{d} = \frac{nV_{sd}}{d} \tag{6}$$

Here d = 0.6 nm is the interlayer spacing, *i.e.* the distance between the tungsten and molybdenum atoms in the heterobilayer. We note that the gate voltage V_g will slightly affect the electric field, but this influence has been included in the n value of the fitting. We have extracted the interlayer electric field in Device 2 from the I-V_{sd} data and show the electric field in the bottom axis of Figure 4b.

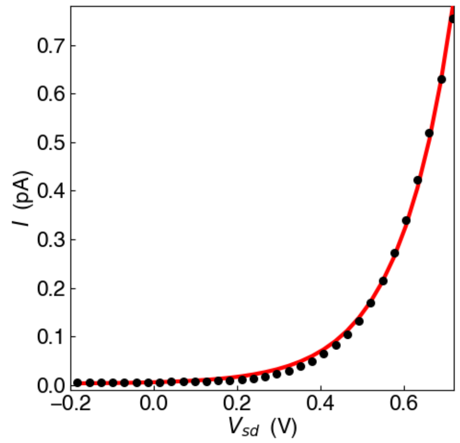

Figure S3 | Extraction of ideality from the dark current of $MoSe_2/WS_2$ heterobilayer. We fit I-V_{sd} data (dots) at $V_g = -6.5\ V$ with Eq. (5) (red line) to find the ideality parameter $n = 0.12$.

3. Estimation of the conduction band minimum offset

By using linear extrapolation based on the Stark effect in Figure 2c (which is presented again in Figure S4a below), we can obtain the interlayer exciton (*IX*) energy to be ~1.69 eV at zero electric field, which is ~90 meV above the intralayer exciton (A_{Mo}) at 1.60 eV. But the 90-meV separation does not represent the offset between the conduction band minima (CBM) of monolayer $MoSe_2$ and WS_2 because the interlayer exciton (*IX*) and the intralayer $MoSe_2$ exciton (A_{Mo}) have different binding energies. To obtain the CBM offset, we estimate the *IX* and A_{Mo} binding energies in the follow way.

First, we estimate the binding energy ratio between intralayer and interlayer excitons. A previous work in $MoSe_2/WSe_2$ heterobilayer[7] calculated the binding energies of the interlayer exciton (114 meV) and intralayer WSe_2 exciton (152.6 meV). The ratio between them is 114/152.6 ≈ 75%. Second, we estimate the reduction of intralayer exciton binding energy in heterobilayer compared to in monolayer. According to a previous paper[8], the intralayer exciton binding energy in BN-encapsulated monolayer WSe_2 is 172.1 meV. The additional screening in the heterobilayer therefore reduces the binding energy of the intralayer exciton to 152.6/172.1 ≈ 90%.

We assume similar binding energy ratios in the case of $MoSe_2/WSe_2$ heterobilayer. A previous work[1] determined the intralayer exciton binding energy to be 212.5 meV in BN-encapsulated monolayer $MoSe_2$. By using the 90% ratio, the intralayer $MoSe_2$ exciton binding energy is estimated to be 212.5 meV × 90% ≈ 191 meV in the $MoSe_2/WS_2$ heterobilayer due to the additional screening. By using the 75 % ratio, the interlayer exciton binding energy is estimated to be 191 meV × 75% ≈ 143 meV (Figure S4b). The binding energy difference between intralayer and interlayer excitons is therefore 191 meV – 143

meV = 48 meV.

By using the 90-meV separation between the intralayer and interlayer excitons at zero field, we obtain the CBM offset as 90 meV – 48 meV = 42 meV (Figure S4b). We estimate that the WS_2 CBM is about 42 meV higher than the $MoSe_2$ CBM in our heterobilayer sample. This supports that our $WS_2/MoSe_2$ heterobilayer exhibits the type-I band alignment.

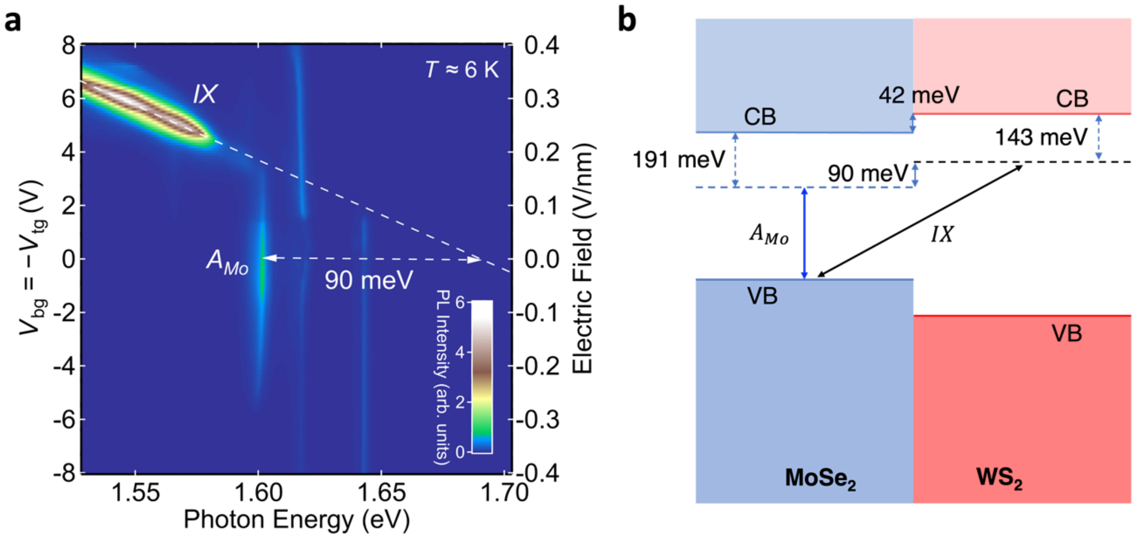

Figure S4 | Estimation of conduction band offset in the $MoSe_2/WS_2$ heterobilayer. a, The electric-field-dependent PL map from Figure 2c. A 90-meV energy separation is extrapolated between interlayer exciton (*IX*) and the intralayer $MoSe_2$ exciton (A_{Mo}) at zero electric field. **b,** Schematic of the band alignment. A band offset of 42 meV is obtained from the binding energies of A_{Mo} (191 meV) and *IX* (143 meV) and the A_{Mo}- *IX* separation (90 meV).

4. Additional results of Device 1: Reflectance contrast measurements

In addition to the PL experiment in the main paper, we have also investigated the absorption properties of Device 1 by reflectance contrast spectroscopy. The reflection experiment is conducted in the Montana cryostat with estimated sample temperature $T \approx$ 6 K (same as the PL experiment). We focus broadband white light onto the sample with a spot diameter of ~2 μm by using an objective lens (numerical aperture 0.6). The reflected light is collected by the same objective and analyzed by a spectrometer (HRS-500-MS, Princeton Instruments) with a charge-coupled-device (CCD) camera. We measure a reflection spectrum (R_s) on the sample and a reference reflection spectrum (R_r) on a nearby area without $MoSe_2$ and WS_2, and obtain the reflectance contrast as $\Delta R/R = (R_s - R_r)/R_r$. We further perform the second-order differentiation on $\Delta R/R$ with respect to the photon energy to bring out the weak features.

Figure S5a, c display the charge-density-dependent reflectance contrast map ($\Delta R/R$) and its second-order energy derivative map [$d^2(\Delta R/R)/dE^2$]. We observe the $MoSe_2$ intralayer exciton A_{Mo} and exciton polarons (A^-_{Mo}, A^+_{Mo}). Figure S5b, d display the electric-field-dependent reflectance contrast map and its second-order energy derivative map. The intralayer exciton A_{Mo} line is prominent in most of the electric-field range and exhibits

little Stark shift. This is reasonable because the intralayer exciton has strong oscillator strength and essentially no interlayer electric dipole.

The interlayer exciton (IX) is not observed in the reflectance contrast maps because it has very weak oscillator strength due to the electron-hole separation in different layers. However, the interlayer exciton state does not leave us no trace of its existence in the reflectance contrast maps. In a narrow region near V_{bg} = - V_{tg} = 4 V, the A_{Mo} line is broken and slightly displaced (marked by an arrow in Figure S5d). This is the same region where the A_{Mo} and IX lines approach each other in the PL map in Figure 2c of the main paper. Our result therefore implies significant mixing of the intralayer and interlayer exciton states when they get close to each other. Similar mixing phenomena of the intralayer and interlayer states were also found in other heterostructures[9]. No such disruption of the A_{Mo} line is observed at negative electric field; this is reasonable because the interlayer excitons emerge only at positive electric field.

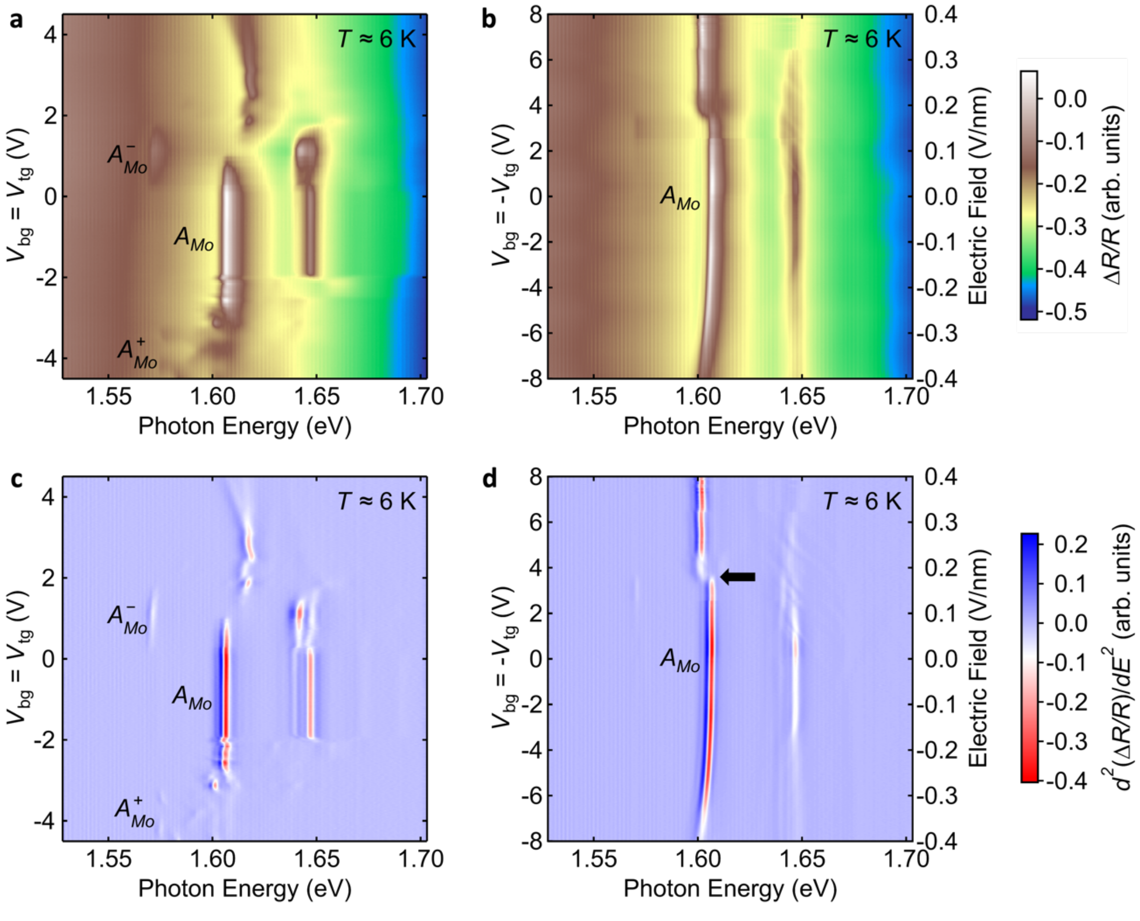

Figure S5 | Gate-dependent reflectance contrast maps and their second-order energy derivatives of $MoSe_2/WS_2$ heterobilayer Device 1. a, The charge-density-dependent reflectance contrast ($\Delta R/R$) map. Equal voltages $V_{bg} = V_{tg}$ are applied to the bottom and top gates. The charge density is proportional to the gate voltages. The $MoSe_2$ intralayer exciton (A_{Mo}) and exciton polarons (A^-_{Mo}, A^+_{Mo}) features are denoted. **b,** The electric-field-dependent reflectance contrast map. Opposite voltages V_{bg} = -V_{tg} are applied to the bottom and top gates to induce an interlayer electric field (right axis). **c,** The second-order energy derivative of the map in panel a [$d^2(\Delta R/R)/dE^2$]. **d,** The second-order energy derivative of the map in panel b. The sample temperature is $T \approx 6$ K.

5. Additional results of Device 2

5.1. Dark current characteristics

Figure 3b in the main paper presents the interlayer current (I) map at varying source-drain voltage (V_{sd}) and gate voltage (V_g) without optical excitation. To better understand the dark current characteristics, Figure S6b presents the cross-cut I-V_{sd} profiles at three selective V_g values. The current is quenched at negative V_{sd}, but it turns on exponentially at positive V_{sd}. In our experiment, the $MoSe_2$ layer is grounded, and V_{sd} is applied on the WS_2 layer. A negative (positive) V_{sd} will therefore raise (lower) the WS_2 conduction band minimum (CBM), while the $MoSe_2$ CBM remains unchanged. The quenching (increasing) current behavior at negative (positive) V_{sd} therefore indicates that the WS_2 CBM is higher than the $MoSe_2$ CBM at zero electric field. This supports our assignment of type-I band alignment in the intrinsic $MoSe_2/WS_2$ heterobilayer.

Figure S6c displays the cross-cut I-V_g profiles at three selective V_{sd} values. Figure S6d presents the corresponding derivative of the logarithmic current with respect to V_g. In Figure S6d, the maxima at $V_g \approx -2$ V signify a crossover from the charge-neutral regime to the n-doping regime. This indicates that the photocurrent measurement in Figure 4 (conducted at $V_g \approx -6.5$ V) is well inside the charge neutral regime.

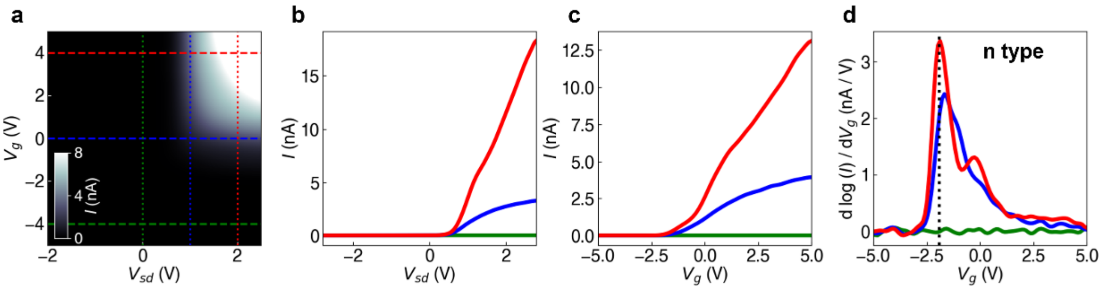

Figure S6 | Dark current characteristics of $MoSe_2/WS_2$ heterobilayer Device 2. a, Interlayer current (I) map as a function of source-drain voltage V_{sd} and gate voltage V_g in the absence of illumination. **b,** Cross-cut I-V_{sd} profile at three selective V_g marked by the horizontal dashed lines in panel a. **c,** Cross-cut I-V_g profile at three selective V_{sd} marked by the vertical dashed lines in panel a. **d,** Derivative of the logarithmic current with respect to V_g in panel c. The maxima at $V_{sd} \approx -2\ V$ signify a crossover from the charge-neutral regime to the n-doping regime.

5.2 Comparison of heterobilayer and monolayer photocurrent response

We have measured the spatial photocurrent (I_{pc}) maps of Device 2 over a range of V_{sd} and V_g by Multiple Parameter Dynamic Photoresponse Microscopy (MPDPM)[10]. Figure S7 displays the I_{pc}-V_{sd} profiles on the $MoSe_2/WS_2$ heterobilayer region and the monolayer $MoSe_2$ region of Device 2, which are marked by the black and blue dots in Figure S7f. The bilayer I_{pc}-V_{sd} profile exhibits a dip around V_{sd} = 1.3 V, indicating suppression of photocurrent, whereas the monolayer profile exhibits monotonically increasing photocurrent. The result confirms that the photocurrent suppression occurs in the heterobilayer.

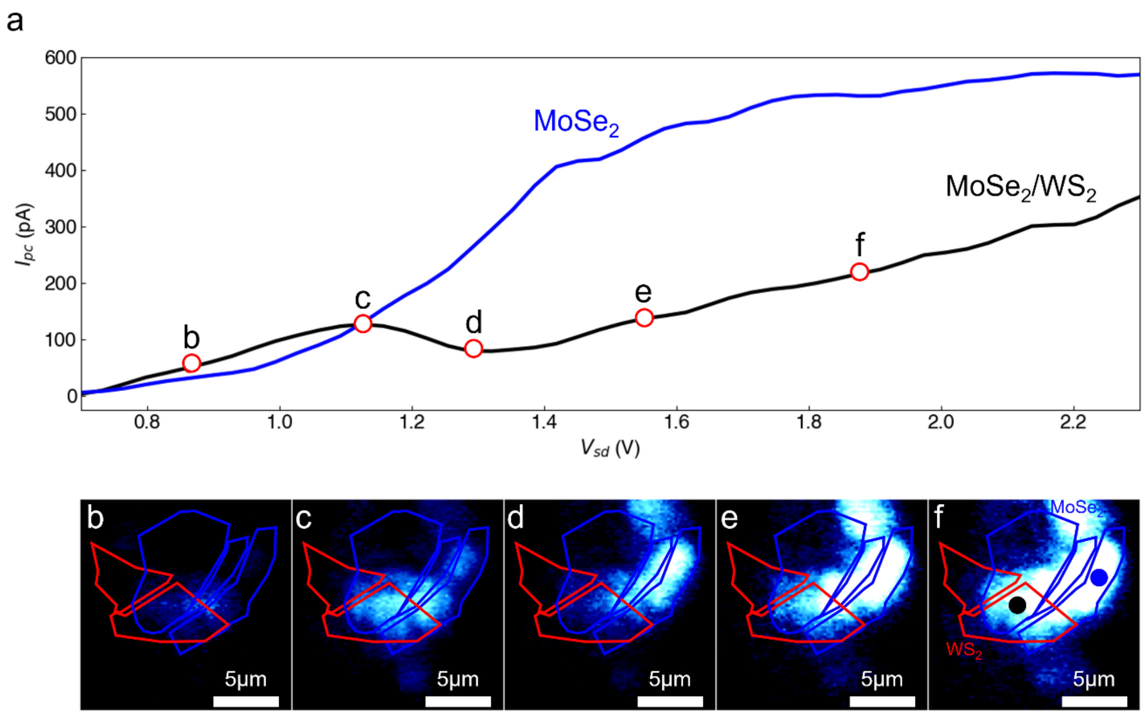

Figure S7 | Comparison between the photocurrent response in the monolayer $MoSe_2$ and heterobilayer regions of Device 2. a, Monolayer $MoSe_2$ (blue) and $MoSe_2/WS_2$ heterobilayer (black) photocurrent at increasing V_{sd}. The photocurrent curves are extracted from the spatial maps in b-f. **b-f,** Spatial photocurrent maps at different V_{sd} and constant V_g. $MoSe_2$ and WS_2 flakes are outlined in blue and red respectively. The black and blue dots in panel f mark the position where the photocurrent profiles in panel a are measured.

6. Results of Device 3: Reproducing interlayer exciton PL

In the main paper, we have presented the PL results of $MoSe_2/WS_2$ heterobilayer Device 1 in Figure 2. Here we also present the PL results of another BN-encapsulated dual-gate $MoSe_2/WS_2$ heterobilayer (Device 3) to show the reproducibility of our main observations. Figure S8 displays the charge-density-dependent PL map and electric-field-dependent PL map of Device 3. We observe the intralayer exciton (A_{Mo}) line with no Stark shift and the interlayer exciton (IX) line with finite Stark shift. The results are similar to those of Device 1 in Figure 2, but all PL features in Device 3 are broader, presumably due to the lower device quality. For instance, the A_{Mo} line width is 10 meV in Device 3, compared to the line width of 3.5 meV in Device 1; the IX line width is 20 meV in Device 3, compared to the line width of 15 meV in Device 1.

We note that the IX Stark shift appears to be somewhat nonlinear in Device 3 (Figure S8b), in comparison to the more linear Stark shift observed in Device 1 (Figure 2c). In general, Stark shift is only strictly linear in the weak field limit. When the electric field is strong, it can distort the exciton wavefunction and cause nonlinear Stark shift. Such distortion of the interlayer exciton wavefunction might depend on the sample quality (e.g. the interface quality), which may account for the slightly different Stark shifts between Device 1 and 3.

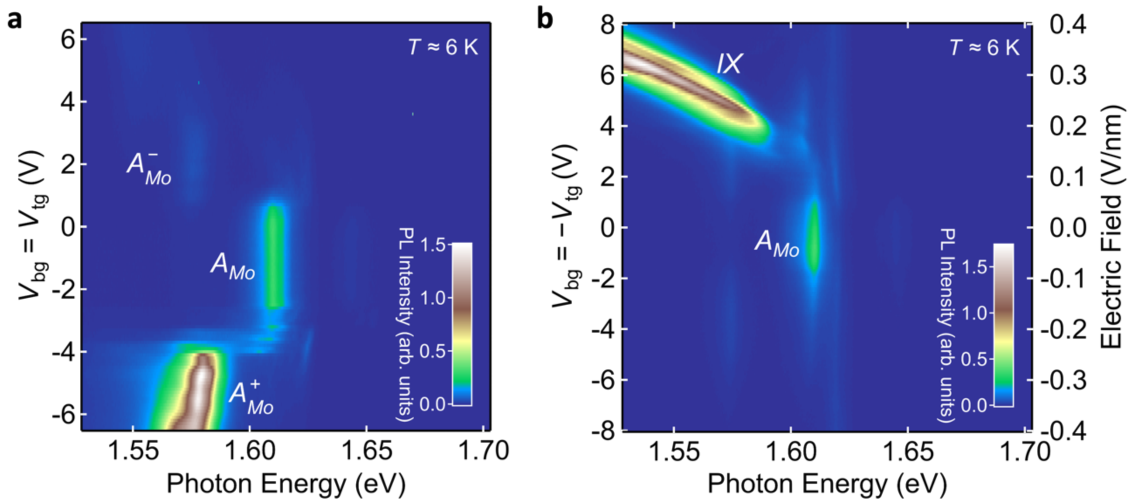

Figure S8 | Gate-dependent photoluminescence (PL) maps of $MoSe_2/WS_2$ heterobilayer Device 3. a, The charge-density-dependent PL map. Equal voltages $V_{bg} = V_{tg}$ are applied to the bottom and top gates. The charge density is proportional to the gate voltages. The A_{Mo}, A^-_{Mo}, A^+_{Mo} features arise from the intralayer excitons, electron-side and hole-side exciton polarons (or trions) in the $MoSe_2$ layer, respectively. **b,** The electric-field-dependent PL map. Opposite voltages $V_{bg} = -V_{tg}$ (left axis) are applied to the bottom and top gates to induce an interlayer electric field (right axis). An interlayer exciton (IX) feature appears at high positive electric field. The measurements were performed with 532-nm continuous laser excitation (incident power ≈ 3 μW) at sample temperature $T \approx 6$ K, the same conditions for Device 1 in Figure 2.

7. Results of Device 4: Photoluminescence and photocurrent

In the main paper, we have presented the PL results in dual-gate Device 1 and photocurrent results in single-gate Device 2. To consolidate the connection between these two types of results, we have fabricated Device 4 and measured both PL and photocurrent on the same device. Device 4 is a BN-encapsulated $MoSe_2/WS_2$ heterobilayer device, which not only has top and bottom graphite gates but also have the source contact on the WS_2 layer and the drain contact on $MoSe_2$ layer (Figure S1d). This complete device setting allows us to measure both PL and photocurrent under an interlayer electric field. Below we will show that the major PL and photocurrent results in Devices 1 and 2 can be reproduced in Device 4.

7.1. PL measurements

Figure S9a-b displays the charge-gate-dependent PL map and electric-field-dependent PL map of Device 4. We observe the intralayer exciton (A_{Mo}), which does not shift under an interlayer electric field. At strong positive electric field, we observe the emergence of the interlayer exciton (IX), which exhibits Stark shift. The IX feature is not observed at negative electric field. These observations are consistent with the results in Figure 2 for Device 1.

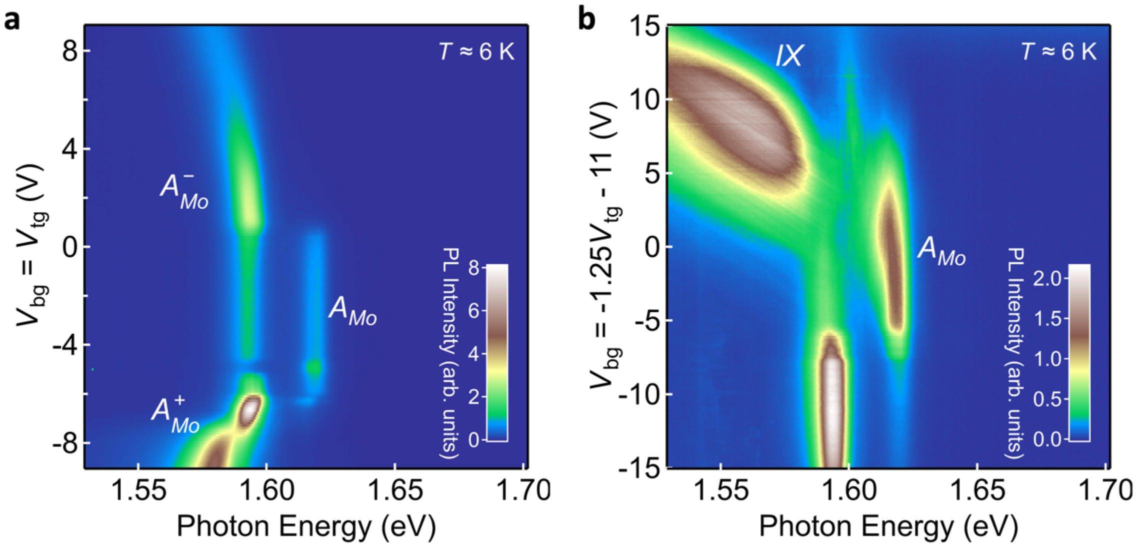

Figure S9 | Gate-dependent photoluminescence (PL) maps of $MoSe_2$/WS_2 heterobilayer Device 4. **a,** The charge-density-dependent PL map. Equal voltages $V_{bg} = V_{tg}$ are applied to the bottom and top gates. The charge density is proportional to the gate voltages. **b,** The electric-field-dependent PL map. Opposite gate voltages $V_{bg} = -1.25V_{tg} - 11$ volts are applied to the bottom and top gates to induce an interlayer electric field while keeping the sample approximately charge neutral. An interlayer exciton (*IX*) feature is observed at high electric field, signifying a type-I to type-II band alignment transition in the heterobilayer. The measurements were performed with 532-nm continuous laser excitation (incident power ≈ 3 μW) at sample temperature $T \approx 6$ K, the same conditions for Device 1 in Figure 2.

7.2. Photocurrent measurements

We have measured the transport and photocurrent properties of Device 4 at varying charge density and interlayer bias. In the experiments, we apply equal voltages $V_{bg} = V_{tg}$ on the top and bottom gates to inject carriers into the heterobilayer but induce almost no out-of-plane electric field. Then we apply a source-drain voltage (V_{sd}) on the WS_2 layer while grounding the $MoSe_2$ layer, and measure the interlayer current. Figure S10a displays the interlayer current map without optical excitation. Figure S10b displays the interlayer current map under 790-nm pulsed laser excitation. These two maps are qualitatively similar to the results of Device 2 in Figure 3b-c. We also plot a cross-cut I_{pc}-V_{sd} profile in Figure S10c. The profile displays a drop of photocurrent at increasing V_{sd}, consistent with the result of Device 2 in Figure 3d. Therefore, the results show that the photocurrent suppression exists both in devices with single silicon gate and dual graphite gates.

We have also measured the photocurrent of Device 4 at different excitation laser power at temperature $T = 50$ K. Here we tune the interlayer electric field with the dual graphite gates rather than the source-drain voltage (V_{sd}). Figure S11a displays the photocurrent as a function of interlayer electric field, which is controlled by top and bottom gates, at increasing excitation laser power. The inset shows the power-dependent photocurrent, from which we can extract the $\gamma/(\alpha+\beta)$ value by using the same analysis method in Figure 4. Figure S11a displays the best-fit $\gamma/(\alpha+\beta)$ value at varying interlayer electric field. We observe a prominent increase of the $\gamma/(\alpha+\beta)$ value starting near $E = 0.13$ V/nm. This signifies the formation of interlayer excitons, consistent with the results in Figure 4 for

Device 2, which controls the electric field via V_{sd}. In summary, both the PL and photocurrent results in Device 4 are consistent with those in Devices 1 and 2.

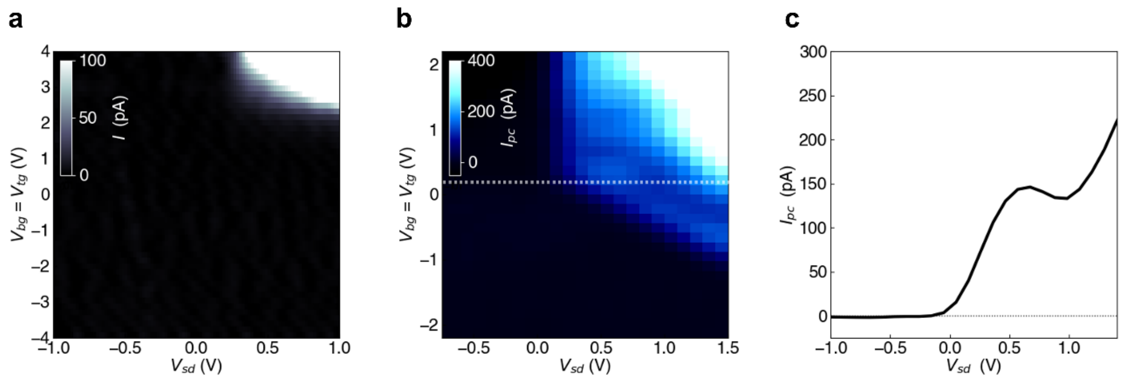

Figure S10 | Photocurrent characteristics of Device 4. a, Dark current map. Equal voltages $V_{bg} = V_{tg}$ are applied on the top and bottom gate. **b,** Photocurrent map under 790-nm pulsed laser excitation, **c,** Cross-cut photocurrent profile along the dashed line in panel b. The measurements were conducted at room temperature.

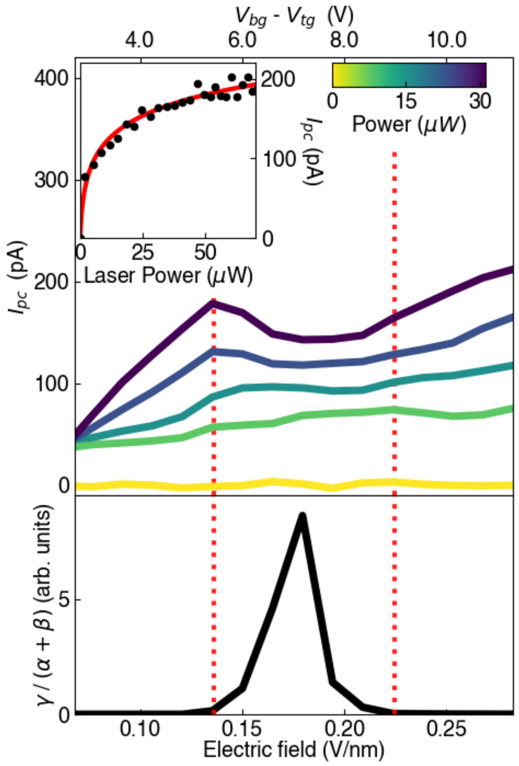

Figure S11 | Photocurrent signature of type-I to type-II band alignment transition in $MoSe_2/WS_2$ heterobilayer Device 4. a, Photocurrent (I_{pc}) as a function of gate voltage difference V_{bg} - V_{tg} (top axis) and the corresponding interlayer electric field (E) (bottom axis) at increasing laser power. We maintain a constant finite charge density in the heterobilayer by keeping $V_{bg} + V_{tg} = 0.85$ V. The inset shows the photocurrent with increasing laser power at a constant interlayer electric field $E = 0.185$ V/nm. The red line is a fit based on the model described in the main paper. **b,** The best-fit $\gamma/(\alpha + \beta)$ value as a function of interlayer electric field. The region between the two vertical dashed lines approximately defines the region where the interlayer excitons are formed. The measurements were conducted with 790-nm pulsed laser at temperature $T = 50$ K.

Supplementary References

1. Liu, E. *et al.* Exciton-polaron Rydberg states in monolayer $MoSe_2$ and WSe_2. *Nat Commun* **12**, 1–8 (2021).
2. Wang, Z., Shan, J. & Mak, K. F. Valley- and spin-polarized Landau levels in monolayer WSe_2. *Nat Nanotechnol* **12**, 144–149 (2017).
3. Altaiary, M. M. *et al.* Electrically Switchable Intervalley Excitons with Strong Two-Phonon Scattering in Bilayer WSe_2. *Nano Lett* **22**, 1829–1835 (2022).
4. Huang, X. *et al.* Correlated insulating states at fractional fillings of the WS_2/WSe_2 moiré lattice. *Nat Phys* **17**, 715–719 (2021).
5. Regan, E. C. *et al.* Mott and generalized Wigner crystal states in WSe_2/WS_2 moiré superlattices. *Nature* **579**, 359–363 (2020).
6. Barati, F. *et al.* Hot carrier-enhanced interlayer electron-hole pair multiplication in 2D semiconductor heterostructure photocells. *Nat Nanotechnol* **12**, 1134–1139 (2017).
7. Liu, E. *et al.* Signatures of moiré trions in $WSe_2/MoSe_2$ heterobilayers. *Nature* **594**, 46–50 (2021).
8. Liu, E. *et al.* Magnetophotoluminescence of exciton Rydberg states in monolayer WSe_2. *Phys Rev B* **99**, 205420 (2019).
9. Tang, Y. *et al.* Tuning layer-hybridized moiré excitons by the quantum-confined Stark effect. *Nat Nanotechnol* **16**, 52–57 (2021).
10. Arp, T. B. & Gabor, N. M. Multiple parameter dynamic photoresponse microscopy for data-intensive optoelectronic measurements of van der Waals heterostructures. *Review of Scientific Instruments* **90**, 023702 (2019).
